# Laryngeal recurrence sites in patients previously treated with transoral laser microsurgery for squamous cell carcinoma

**DOI:** 10.1186/s40463-018-0266-y

**Published:** 2018-02-13

**Authors:** P. Horwich, M. H. Rigby, C. MacKay, J. Melong, B. Williams, M. Bullock, R. Hart, J. Trites, S. M. Taylor

**Affiliations:** 1 Department of Surgery, Division of Otolaryngology–Head and Neck Surgery, Queen Elizabeth II Health Science Centre and Dalhousie University, 3rd Floor Dickson Building, VG Site, 5820 University Avenue, Halifax, NS B3H 2Y9 Canada; 2 Department of Pathology, Division of Anatomical Pathology, Queen Elizabeth II Health Science Centre and Dalhousie University, Halifax, NS Canada

**Keywords:** Laryngeal Recurrence, Transoral Laser Microsurgery, Squamous Cell Carcinoma

## Abstract

**Background:**

The laryngeal framework provides a natural barrier preventing tumour spread to extralaryngeal structures. Transoral laser microsurgery (TLM) for laryngeal squamous cell carcinoma (SCC) may violate these boundaries, altering the pathways of tumor spread for potential recurrences. Our project objective is to describe laryngeal SCC recurrence patterns and overall survival in patients requiring total laryngectomy (TL) after TLM.

**Methods:**

Patients undergoing TLM for laryngeal SCC requiring salvage TL were identified from a prospective CO2 laser database containing all patients undergoing TLM for head and neck malignancies at the QEII Health Sciences Center in Halifax, Nova Scotia between March 2002 – May 2014. Surgical pathology reports were analyzed for tumor characteristics, extent of recurrence and invasion of local structures. Kaplan-Meier analyses were performed to evaluate overall survival, disease specific survival (DSS) and locoregional control.

**Results:**

Fifteen patients were identified from the database as receiving salvage TL for recurrent disease after initial TLM resection for laryngeal SCC. Final pathology reports demonstrated that 67% (10/15) of patients had thyroid cartilage involvement while 53% (9/15) of patients had cricoid cartilage involvement on salvage TL pathology. 33% (5/15) of patients had perineural invasion and 27% (4/15) had lymphovascular invasion. Mean and median follow-up times were 36.7 months and 26.8 months respectively (range 3.9–112.6). The Kaplan-Meier estimate for overall survival at 36 months was 40% post TL with a standard error (SE) of 13.6%. DSS was 47% (SE 14.2%), and locoregional control was 55% (SE 14.5%) post TL.

**Conclusions:**

Laryngeal recurrence sites following TLM seem to be consistent with historical data at known laryngeal sites of vulnerability. Treatment with TLM does not predispose patients to a lower rate of locoregional control and overall survival after total laryngectomy and salvage outcomes are consistent with literature values.

## Background

Treatment for laryngeal squamous cell carcinoma (SCC), even for advanced disease, has shifted from radical laryngectomies in favour of organ preservation therapies. Transoral laser microsurgery (TLM) is an example of one such organ preserving technique. TLM involves resecting the tumour in pieces as opposed to en bloc tumour resection, thereby allowing for preservation of surrounding structures. TLM has been shown to be an effective alternative to open surgical techniques with comparable or even superior local control for both supraglottic and glottic SCC primary tumours [1, 2]. Dalhousie University has a well-established TLM program, and we have published multiple studies on laryngeal SCC outcomes and cost-effectiveness of TLM treatment [1–4]. Locoregional control at 2 years for early glottic cancer treated with TLM has been shown to be 95% [3]. TLM has become the treatment modality of choice for all early, and some advanced cases of laryngeal SCC at our center. However, there are concerns that TLM and other minimally invasive techniques may alter the natural laryngeal framework, predisposing patients to future recurrences and atypical tumour spread.

The laryngeal framework contains various cartilaginous and ligamentous structures, such as the conus elasticus, that provide natural anatomic barriers to tumour spread. Initially published by Kirchner in 1976, these framework studies also mapped out paths of least resistance for tumour spread, such as through the pre-epiglottic space and paraglottic spaces. This allows clinicians to have a sense of predictability for tumour spread and is the basis of both modern tumour staging guidelines and oncologic surveillance for recurrent disease.

The violation or compromise of these natural anatomic barriers of the laryngeal framework may lead to atypical tumour spread through perichondrial defects potentially facilitating early extralaryngeal spread and ultimately impacting patient survival. It is unclear from the literature if previous treatment with TLM alters the pathways for tumour spread in the larynx.

The purpose of this study is to determine if TLM of laryngeal SCC alters the natural barriers of the larynx leading to atypical sites of recurrence and to evaluate if TLM predisposes patients to lower rates of locoregional control and survival.

## Methods

This is a case series based on a center specific CO2 laser database containing all patients undergoing TLM for head and neck malignancies at the QEII Health Sciences Center in Halifax, Nova Scotia. Our TLM database is prospectively maintained and contains over 300 patients up to the publication date. All patients undergoing salvage total laryngectomy with a previous history of TLM treatment from April 2002 – March 2014 were identified. Baseline patient characteristics, pre-operative tumour stage, pathology report information and follow-up data were collected from the database proper. Pathology reports were all signed off by our local head and neck pathologist (MB). Our standardized, center specific pathological reporting for total laryngectomy specimens was followed that includes data on laryngeal subsites, areas of cartilage or bone involvement and areas of extra-laryngeal extension. Ethics approval for the study was obtained from the Nova Scotia Health Authority Research Ethics Board (ROMEO #1020643).

Kaplan-Meier 36-month survival analyses were performed using SPSS for the following endpoints: overall survival (OS) post TL, disease-specific survival post TL, and locoregional control post TL.

## Results

From the database, 15 patients were identified to have biopsy confirmed SCC of the larynx initially treated with TLM who required salvage TL for recurrent disease. Five out of 15 patients were found to have two TLM treatments prior to proceeding to total laryngectomy. The majority of patients were male (93%) with a mean age of 66 (range 48–64). Baseline patient characteristics are illustrated in Table 1.

**Table 1:** Baseline Patient Characteristics

Patient Characteristics	Value
Male	14/15 (93%)
Mean Age (range)	66.5 (48–94)
History of Smoking	6/15 (40%)
Radiation Prior to TL	9/15 (60%)
Initial Laser to TL in Months (range)	19.6 (2–87)
TLM with Salvage Radiation	9/15 (60%)

Thirteen patients had a glottic primary site while two patients had a supraglottic primary site. Both supraglottic primaries were centered on the left arytenoid cartilage with extension to the infra-hyoid epiglottis. Tumour stage on initial presentation varied from T1a (7%) to T3 (47%) (Table 2).

**Table 2:** TNM Staging on Initial Presentation

Tumor Stage	Value
T1AN0M0	1/15 (7%)
T2N0M0	7/15 (47%)
T3N0M0	6/15 (40%)
T3N1M0	1/15 (7%)

**Fig. 1:**
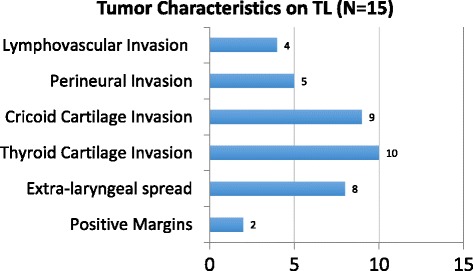
Pathology Tumour Characteristics on Total Laryngectomy

Lymphovascular and perineural invasion was found in 27% (4/15) and 33% (5/15) of patients respectively. Cricoid cartilage invasion was found in 60% (9/15) of specimens while thyroid cartilage invasion was found in 67% (10/15) of specimens. (Fig. 1).

**Fig. 2:**
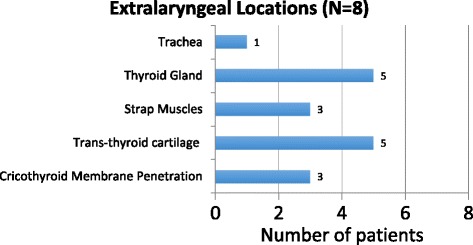
Extralaryngeal Extension Locations on Pathology

Extra-laryngeal extension was present in 53% (8/15) of patients. Of those with extra-laryngeal extension, 62% (5/8) had tumour penetration through the thyroid cartilage and similarly 62% (5/8) had thyroid gland invasion. Tracheal involvement was found in 12% (1/8) of patients, while strap muscle involvement and cricothyroid membrane penetration were found in 37% (3/8) of patients. (Fig. 2).

Mean and median follow-up times were 36.7 months and 26.8 months respectively (range 3.9–112.6). The Kaplan-Meier estimate for OS was 40% (SE 13.6%) at 36 months post TL. The Kaplan-Meier estimate for disease specific survival (DSS) was 47% (SE 14.2%) at 36 months post TL. The Kaplan-Meier estimate for locoregional control was 55% (SE 14.5%) at 36 months post TL.

## Discussion

Previous anatomical studies have demonstrated that the natural laryngeal framework provides a predictable pathway for tumour spread [5–9]. Physical barriers to tumour spread in the larynx include the conus elasticus, and the perichondrium of the thyroid cartilage. With the introduction of minimally invasive techniques such as TLM, there are concerns that this natural framework may be altered, increasing the risk of atypical tumour spread and ultimately tumour recurrence. Our current study is the first in the literature to address this issue, by reviewing the pathology of tumour spread in patients who required a salvage TL after initial TLM resection.

Our study demonstrated that despite initial TLM resection, patients who developed a laryngeal recurrence did so in a similar predictable manner as demonstrated in previous anatomical studies [5–9]. The pre-epiglottic space and paraglottic spaces are known to contain fibrofatty tissue and multiple lymphatic channels, providing a path of least resistance for tumour spread [5]. The pre-epiglottic space is bound by the epiglottis posteriorly, the thyroepiglottic ligament inferiorly and the hyoepiglottic ligament superiorly. The pre-epiglottic space communicates with the bilateral paraglottic spaces, which are bound laterally by the perichondrium of the thyroid cartilage, posteriorly by the mucosa of the pyriform sinus and medially by either the quadrangular membrane superior, or the conus elasticus inferior to the laryngeal ventricle.

**Fig. 3:**
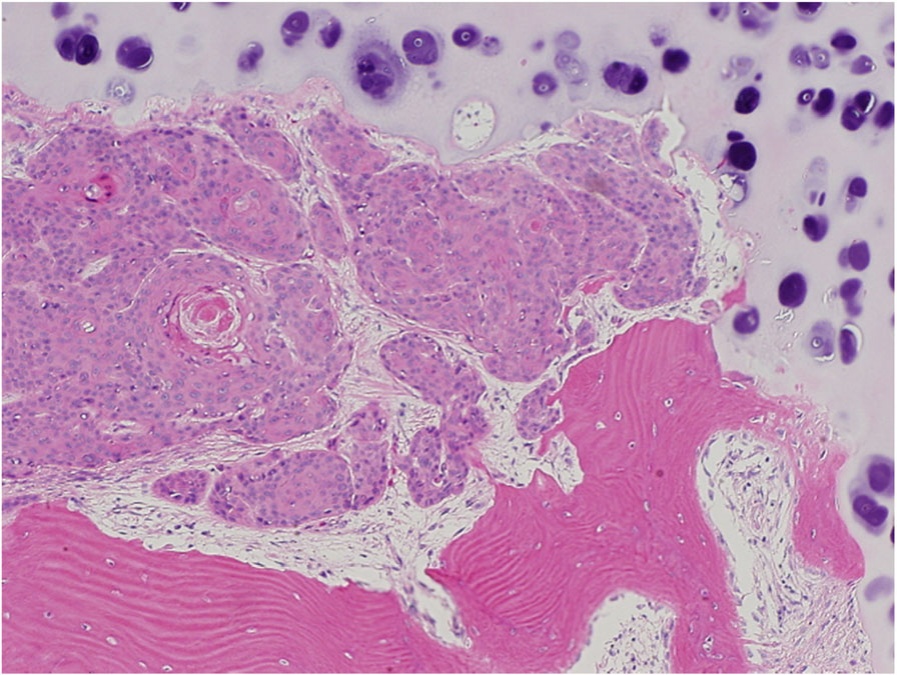
Squamous cell carcinoma recurrence infiltrating thyroid cartilage at top of slide

**Fig. 4:**
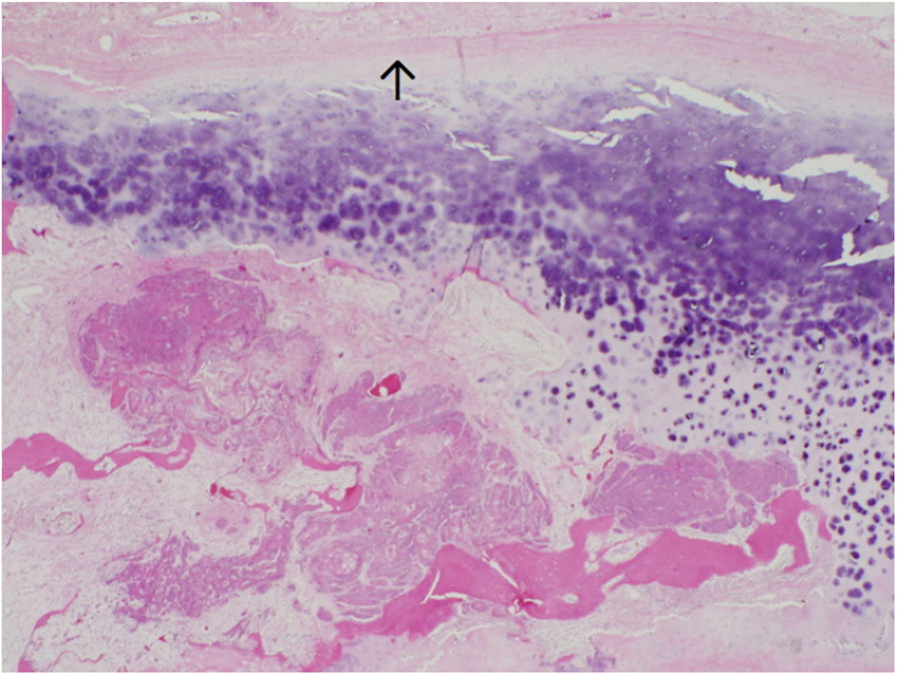
Squamous cell carcinoma invading medial thyroid perichondrium, thyroid cartilage. Anterior perichondrium intact, indicated by arrow

Tumour spread after TLM in our study occurred most frequently by paraglottic extension into the cricoid and thyroid cartilage. Similarly, among patients with extra-laryngeal extension, tumour spread occurred most commonly through the thyroid cartilage (Fig. 3) and cricothyroid membrane, in keeping with known laryngeal framework vulnerabilities and tumour spread patterns described by Kirschner. The perichondrium of the thyroid cartilage is an important barrier to prevent direct extralaryngeal extension [6, 7]. As shown in Fig. 4, the medial perichondrium and thyroid cartilage have been invaded however the anterior perichondrial layer remains intact.

Interestingly, 5/15 (33%) patients had direct thyroid gland involvement on pathology review in our study – this is significantly higher than literature values. A recent systematic review and meta-analysis including 1180 patients by Kumar et al. noted a thyroid gland involvement rate of 10.7% in patients undergoing total laryngectomy [10]. A 2015 publication from Mourad et al. reported thyroid gland involvement in 2.7% of 343 patients undergoing primary or salvage total laryngectomy [11]. Our results may indicate that previous TLM treatment may be an indication for thyroidectomy during total laryngectomy however with such a small sample size further study on this subject is required.

Our study also demonstrates that patients who undergo initial TLM resection for laryngeal SCC are not at an increased risk of locoregional recurrence or have a decrease in DSS after salvage TL when compared to patients undergoing primary TL. At 36 months, our patient population’s DSS was found to be 47% — similar to previous studies reporting DSS between 50 and 60% for laryngeal SCC treated with primary TL [9, 12, 13].

Previous studies have demonstrated that radiation may induce changes to the laryngeal framework at the molecular level, possibly increasing the risk of tumour invasion [14, 15]. Five patients received primary RT prior to TLM and subsequent TL. In the current study, 10 patients (66%) had postoperative radiation therapy (after TLM), prior to salvage TL. As a result, postoperative radiation may have caused changes to the laryngeal framework demonstrated in the final pathology. Zbaren et al. demonstrated that patients with primary radiation failures requiring salvage TL had statistically increased tumor multifocality versus concentric growth patterns found in primary laryngeal cancers treated with total laryngectomy [15]. Given our study had maintained predicted laryngeal patterns of tumour spread, it is unlikely that radiation therapy had a significant impact on our results.

Our study is not without limitations. One of the major limitations of our study is the relatively small sample size. In order for tumour spread after TLM to be adequately characterized, pathology of the entire larynx needed reviewing. This limited our inclusion criteria to only those patients who underwent a salvage TL for locoregional recurrence after initial TLM resection. Previous studies have also demonstrated that different subsite involvement can affect tumour spread and extralaryngeal involvement [5–8]. With our limited sample size, it is difficult for us to draw overall conclusions with respect to this.

## Conclusion

Laryngeal recurrence sites following TLM seem to be consistent with historical data at known laryngeal sites of vulnerability. Treatment with TLM does not predispose patients to a lower rate of locoregional control and overall survival is consistent with literature values.

## Abbreviations

DSS: Disease Specific Survival
OS: Overall Survival
RT: Radiation Treatment
SCC: Squamous Cell Carcinoma
SE: Standard Error
TL: Total Laryngectomy
TLM: Transoral Laser Microsurgery
